# Supplementary material for: Dynamic transcriptomic profiles of zebrafish gills in response to zinc supplementation
Source: BMC Genomics. 2010 Oct 11;11:553. doi: 10.1186/1471-2164-11-553 (PMC3091702; doi:10.1186/1471-2164-11-553)
Supplement: Additional file 2 — Interactive Direct Interaction Network representing the molecular interactions between zinc, copper, iron, calcium and proteins encoded by transcripts changed by zinc supplementation. Mini web-site containing index.html and hyperlinked pages in subdirectory describing a Direct Interaction Network automatically generated based on curated interactions contained within the proprietary PathwayArchitect database. Ovals represent proteins and the circles symbolize metal ions. Objects are coloured by their abundance in zebrafish at the time-point they were significantly different from the control is a scale from -4 fold (dark green) to +4 fold (dark red). Where significant differences were found at more than one time-point, the colour overlay shows expression at the first instance. Dark blue squares denote 'binding', and light blue squares 'expression'; green squares stand for 'regulation', green diamonds for 'metabolism', and green circles for 'promoter binding'. Arrow heads indicate directionality of the interaction where annotated. All nodes and edges can be further interrogated by selecting the relative area of the image. [file 1471-2164-11-553-S2.zip › PathwayArchitect Zn xs DIN/564248.html]

# REGULATION:

|  |  |
| --- | --- |
| Type | REGULATION |
| Effect | Positive |


---

|  |  |
| --- | --- |
| Score | 0 |


---

|  |  |
| --- | --- |
| Reference Count | 8 |


---

|  |  |
| --- | --- |
| Mechanism | Unknown |


---

|  |  |
| --- | --- |
| Reference:0 || Sentence | "A requirement for Stat1 binding to ISRE for IFN-gamma and IFN regulatory factor-1 (IRF-1) binding to ISRE for LPS, poly(I:C), and virus has been reported." |
| PMID | 9858321 |
| Year | 1998 |
| Species | Human |
|  | Rat |
| Journal | J Interferon Cytokine Res |
| RefScore | 0 |
| Source | PArchNLP |
  |
|  |  |


---

|  |  |
| --- | --- |
 Reference:1 || Sentence | "Induction of IRF-1 by IFNgamma requires functional STAT1 signaling and was abrogated by butyrate, trichostatin A, suberoylanilide hydroxamic acid, and STAT1 small interfering RNA." |
| PMID | 15123634 |
| Year | 2004 |
| Species | Human |
| Journal | J Biol Chem |
| RefScore | 0 |
| Source | PArchNLP |
  ||  |  |


---

|  |  |
| --- | --- |
 Reference:2 || Sentence | "The enhanced activation of STAT1 resulted in increased induction of mRNA encoding interferon regulatory factor-1." |
| PMID | 10477259 |
| Year | 1999 |
| Species | Mouse |
| Journal | Biochem J |
| RefScore | 1 |
| Source | PArchNLP |
  ||  |  |


---

|  |  |
| --- | --- |
 Reference:3 || Sentence | "Taken together, our results suggest that CAF activates STAT1, leading to IRF-1 induction and inhibition of gene expression regulated by the HIV-1 LTR." |
| PMID | 11752148 |
| Year | 2001 |
| Species | Human |
| Journal | J Virol |
| RefScore | 0 |
| Source | PArchNLP |
  ||  |  |


---

|  |  |
| --- | --- |
 Reference:4 || Sentence | "For VSV infection, IFN-gamma signals through Jaks 1 and 2 and STAT1 to activate (interferon regulatory factor) IRF-1; although viral protein synthesis is inhibited, PKR is not a critical mediator in the antiviral response to VSV in murine neurons." |
| PMID | 12401479 |
| Year | 2002 |
| Species | Human |
| Journal | Cytokine Growth Factor Rev |
| RefScore | 1 |
| Source | PArchNLP |
  ||  |  |


---

|  |  |
| --- | --- |
 Reference:5 || Sentence | "Influenza A virus more efficiently activated ISGF3 complex formation and Stat1 DNA-binding compared to Sendai virus, which in turn was a more potent activator of IRF-1." |
| PMID | 11022002 |
| Year | 2000 |
| Species | Human |
| Journal | Virology |
| RefScore | 1 |
| Source | PArchNLP |
  ||  |  |


---

|  |  |
| --- | --- |
 Reference:6 || Sentence | "Despite the presence of functional STAT1, IRF-1 and USF-1, activators implicated in CIITA expression, IFN-gamma did not induce CIITA in cells lacking BRG1 and hBRM, the ATPase subunits of SWI/SNF." |
| PMID | 11953317 |
| Year | 2002 |
| Species | Human |
| Journal | EMBO J |
| RefScore | 0 |
| Source | PArchNLP |
  ||  |  |


---

|  |  |
| --- | --- |
 Reference:7 || Sentence | Induction of IRF-1 by IFNgamma requires functional STAT1 signaling and was abrogated by butyrate, trichostatin A, suberoylanilide hydroxamic acid, and STAT1 small interfering RNA. |
| Year | 2004 |
| PMID | 15123634 |
| Species | Human |
| Journal | J Biol Chem |
| RefScore | 2 |
| Source | PArchNLP |
  |


---

|  |  |
| --- | --- |
